# Variation of fatty acid desaturation in response to different nitrate levels in *Auxenochlorella pyrenoidosa*

**DOI:** 10.1098/rsos.181236

**Published:** 2018-11-28

**Authors:** Qi Zhang, Zaizhi You, Xiaoling Miao

**Affiliations:** 1 State Key Laboratory of Microbial Metabolism, School of Life Sciences and Biotechnology, Shanghai Jiao Tong University, 800 Dongchuan Road, Shanghai 200240, China; 2 Biomass Energy Research Center, Shanghai Jiao Tong University, Shanghai 200240, China

**Keywords:** *Auxenochlorella pyrenoidosa*, nitrate levels, oleic acid, fatty acid desaturase

## Abstract

Microalgae are promising feedstocks for biodiesel, where the high proportion of monounsaturated fatty acid such as oleic acid (C18:1) is preferred. To regulate fatty acid desaturation in microalgae, the relationship among nitrate concentration, fatty acid composition and the expression levels of desaturase genes was explored. Dynamic variations of fatty acid profiles suggested nitrate could induce desaturation of C18 fatty acids. The content of C18:1 in *Auxenochlorella pyrenoidosa* was 30.88% at 0 g l^−1^ nitrate concentration compared with 0.48% at 1.5 g l^−1^. The expressions of relative delta-9, 12 and 15 fatty acid desaturase genes (Δ9, Δ12 and Δ15FADs) were further investigated. The 330% upregulated expression of Δ9FAD in logarithmic phase at 0 g l^−1^ resulted in C18:1 accumulation. Moreover, nitrate replenishment caused a sharp reduction of C18:1 from 34.79% to 0.22% and downregulation of Δ9FAD expression to 1% of the nitrate absence level, indicating the pivotal role of Δ9FAD in C18:1 accumulation. Finally, overexpression of Δ9FAD in *Escherichia coli* and *Saccharomyces cerevisiae* resulted in an increase of C18:1, confirming its ability of desaturating C18:0. The results could provide a new approach and scientific guidance for the improvement of biodiesel quality and industrialization of high-valued chemicals by means of metabolic engineering.

## 1. Introduction

Globally, the air pollution and climate change caused by combustion of fossil fuel lead to increasing demands of the renewable and sustainable energy [1]. Microalgae biofuels such as methane, biodiesel and biohydrogen have attracted much attention [2]. The biodiesel derived from microalgae is biodegradable and non-toxic, deemed as the third generation biodiesel [3,4]. Biodiesel is a kind of fatty acid methyl esters mixture obtained by transesterification, the quality of which is deeply affected by the chain length and amount of double bonds of fatty acids [5]. Monounsaturated fatty acids like oleic acid (C18:1) are preferred for biodiesel. Such components maintain a good balance between cold flow and oxidative stability [6,7]. In addition, oleic acid has demonstrated activity in cancer prevention [8], thus making it a high-valued product of bioengineering.

The fatty acid composition of microalgae is dramatically influenced by cultivation mode [9], nutrition stress [10] and environmental factors [11]. Numerous studies have focused on the impacts of nutrition stress on the fatty acid composition of microalgae, where nitrogen accounts for the majority [12,13]. Nitrogen starvation is generally considered as the most common and effective way to trigger lipid accumulation for biodiesel purpose, although it generates a concomitant reduction of growth [14]. Nitrogen deprivation greatly increased the content of total fatty acids and polyunsaturated fatty acids (PUFAs) [15]. A study has reported that oleic acid (C18:1) was accumulated only under nitrate absence in *Chlorella pyrenoidosa*, while the content of linoleic acid (C18:2) and linolenic acid (C18:3) increased in the presence of nitrate [16]. Although these studies have been conducted to explore the influence of nitrogen on algal fatty acid composition [17,18], the relationship between nitrogen and fatty acid desaturation metabolism is still unclear.

There are two pathways in the biosynthesis of unsaturated fatty acids: ω3 and ω6, distinguished by the distance between the last double bond and the methyl end of the acyl chain [19,20]. The biosynthesis of long chain PUFA starts with oleic acid, using a set of desaturases and elongases in the smooth endoplasmic reticulum to produce PUFA like EPA and DHA [21]. The production of medium chain fatty acids in *Auxenochlorella pyrenoidosa* involves delta-9, 12 and 15 fatty acid desaturases. Delta-9 fatty acid desaturase (Δ9FAD) catalyses the first step in the PUFA biosynthetic pathway, which triggers the transition from saturated fatty acids to monounsaturated ones [22]. It adds a double bond to acyl chain between carbons 9 and 10 of hexadecanoic acid (C16:0) and stearic acid (C18:0), producing hexadecenoic acid (C16:1) and oleic acid (C18:1), respectively. Subsequently, delta-12 fatty acid desaturase (Δ12FAD) introduces a double bond at the delta-12 position of C18:1 to form C18:2. Further, delta-15 fatty acid desaturase (Δ15FAD) catalyses the conversion from C18:2 to C18:3 [23].

The expressions of these three fatty acid desaturase genes (Δ9FAD, Δ12FAD and Δ15FAD) are influenced by cultivation stress. Δ12FAD was known to participate in adaptation to low temperatures [24]. Δ9FAD played an important role in response to altered salt stress in *Chlamydomonas* sp. ICE-L [25]. The expressions of Δ15FAD and Δ9FAD were stimulated under 150 µmol m^−2^ s^−1^ white LED and 70 µmol m^−2^ s^−1^ blue LED, respectively [26]. However, few studies focused on the impact of nitrogen on the expressions of Δ9, Δ12 and Δ15FADs in microalgae, which are closely related to desaturation of C18 fatty acids.

On the other hand, metabolic engineering is a promising tool to accumulate desirable fatty acids in microalgae, specifically endogenous synthesis of fatty acids [27,28]. However, genetic manipulation requires a comprehensive knowledge of the fatty acid desaturation metabolism. Thus, it is of significance to figure out the impact of nitrate concentration on transcriptional levels of FADs.

In the current study, the dynamic variations of fatty acid profiles were deeply investigated under different initial nitrate concentrations. Moreover, the relationship between nitrate concentration and the transcriptional levels of Δ9, Δ12 and Δ15FADs was also studied. The key Δ9FAD was cloned and overexpressed. We aim to provide a comprehensive understanding of the fatty acid desaturation metabolic pathway in response to different nitrate concentrations, and thus pave the way for accumulating C18:1 by means of manipulating environmental conditions and metabolic engineering.

## 2. Material and methods

### 2.1. Microalgae cultures

*Auxenochlorella pyrenoidosa* was preserved in the modified BG-11 medium. The initial OD_600_ was 0.2. The cultivation of *A. pyrenoidosa* was under 25 ± 1°C and 140 µmol m^−2^ s^−1^ in 1 l Erlenmeyer flask (200 mm length, 100 mm diameter) with 500 ml working volume of modified BG-11 medium. BG-11 medium consists of (per litre) 1.5 g NaNO_3_; 0.03 g K_2_HPO_4_; 0.075 g MgSO_4_ · 2H_2_O; 0.036 g CaCl_2_ · 2H_2_O; 0.006 g citric acid; 0.006 g ferric ammonium citrate; 0.001 g EDTA; 0.02 g Na_2_CO_3_ and 1 ml micronutrient solution. The micronutrient solution consists of (per litre) 2.86 g H_3_BO_3_; 1.81 g MnCl_2_ · 4H_2_O; 0.222 g ZnSO_4_ · 7H_2_O; 0.39 g NaMoO_4_ · 5H_2_O; 0.079 g CuSO_4_ · 5H_2_O; 0.0494 g Co(NO_3_)_2_ · 6H_2_O [29]. BG-11 medium and nitrogen-depleted BG-11 medium were prepared to cultivate *A. pyrenoidosa*. For nitrate replenishment, the initial nitrate concentration in the medium was 0 g l^−1^, nitrate concentration was increased to 1.5 g l^−1^ after 8 days cultivation.

### 2.2. Determination of growth and biomass production

The dry cell weight (g l^−1^) of *A. pyrenoidosa* during the cultivation was carefully measured using the method of Chiu *et al.* [30]. The cells of *A. pyrenoidosa* were harvested by centrifugation (Avanti JE, Beckman, Germany) at 8000 r.p.m. for 10 min and washed twice with distilled water. After precooling, the cells were lyophilized in a freeze drier. A calibration curve of OD_600_ versus cell density was constructed. Cell density was calculated using the equation: cell density (g l^−1^) = 0.2898 × OD_600_ + 0.0276 (*R*^2^ = 0.9944). Therefore, the optical density could be effectively used to represent the resulting biomass production.

### 2.3. Determination of nitrogen concentration

In this study, nitrogen was solely supplied as sodium nitrate. The nitrogen concentration in the medium was measured every day during the cultivation. A 10 ml microalgae culture was first centrifuged (8000 r.p.m., 15 min), and then a 0.45 µm syringe filter was used to filter the supernatant. The determination of nitrate concentration was performed using an automatic chemistry analyser (Smartchem 200, Alliance, France). Three independent samples were collected and measured.

### 2.4. Lipid extraction and fatty acid analysis

Algal cells were harvested by centrifugation every day during the cultivation. The total lipids were extracted using a modified method [31]. Lyophilized algae powder (0.2 g) was pulverized in a mortar and extracted using 10 ml solvent mixture of chloroform : methanol (2 : 1, v/v). After shaking for 15 min, the samples were centrifuged (5804R, Eppendorf, Germany) at 8000 r.p.m. for 10 min. The procedure was repeated three times to make sure the lipids were extracted completely. The solvent phase was transferred by pipette and evaporated in a water bath at 55°C. The fatty acid methyl ester (FAME) composition was analysed after the acidic transesterification of lipid [32]. After reaction for 2 h, 1 ml hexane and 1 ml sodium chloride water solution were added to the sample, then vibrated gently and centrifuged. 1 µl of the organic upper phase was injected into an Auto System XL GC/Turbo Mass MS (Perkin Elmer, Germany) using DB-5MS (5% phenyl)-methylpolysiloxane nonpolar column (30 m × 0.25 mm × 0.25 µm). At the beginning, the column temperature was kept at 60°C for 4 min, and then increased to 220°C, finally reached to 280°C with a temperature gradient of 10°C min^−1^, maintained for 10 min [16].

### 2.5. RNA extraction and cDNA synthesis

Total RNA extraction was performed with Trizol reagent (Sangon Biotech, Shanghai, China). Algal cells were harvested by centrifugation in lag (day 2), logarithmic (day 7) and stationary (day 12) phases under the nitrate concentrations of 0 and 1.5 g l^−1^, respectively. Approximately, algal cell (100 mg) was ground in liquid nitrogen to powder before adding 1 ml Trizol reagent. After centrifugation at 12 000 r.p.m. for 10 min at the temperature of 4°C, 200 µl chloroform was added and mixed thoroughly. Sample was centrifuged at 12 000 r.p.m. for 15 min, and then 450 µl of the uppermost layer was transferred into a fresh tube. Isopropanol (450 μl) was added to precipitate RNA and then centrifuged at 12 000 r.p.m. for 15 min, washed with 1 ml ethanol (75%) twice, dissolved in 30 µl diethyl pyrocarbonate treated distilled water [25].

M-MLV reverse transcriptase (TaKaRa Biotech Co., Dalian, China) was used to synthesize the cDNA. Three fatty acid desaturases (Δ9FAD, Δ12FAD, Δ15FAD) were investigated in this study. Genome annotation identified putative components of the C18 fatty acid biosynthetic pathway including Δ9, Δ12 and Δ15FADs [33]. Primers were designed according to the highly conserved regions obtained from the alignment of public database and the deduced amino acid sequences (electronic supplementary material, figure S1). The primers used in the experiment are shown in table 1. After amplification, the PCR products were purified and cloned into pMD-19T vector for sequencing.

**Table 1.** Primers used in this experiment.

primer name	sequence (5′ to 3′)	purpose
Δ9FAD_qPCR_F	AACCCCTACCTGGGCTTCATCT	quantitative RT-PCR
Δ9FAD_qPCR_R	ATGCGGGTGTAGGCAATCTCG	
Δ12FAD_qPCR_F	ATGCGGGTGTAGGCAATCTCG	
Δ12FAD_qPCR_R	GGGAGGAGACGCTGAAGAAGAG	
Δ15FAD_qPCR_F	CGAGGGCTCCCACTTCGACC	
Δ15FAD_qPCR_R	TCCAGCCACATCACAAACACCC	
β-Actin_F	GCTCAACTCCTCCACGCT	
β-Actin_R	GTCCTTGCGGATGTCCAC	
Δ9FAD_F	GACAAGTTTGCAGAGGAGCAG	gene cloning
Δ9FAD_R	TCAGACGGACACCTCGCGGT	
Δ9FAD_BamHI_F	CGCGGATCCATGGACAAGTTTGCAGAGGAGCAG	construction of expression vector for *E. coli*
Δ9FAD_HindIII_R	CCCAAGCTTTCAGACGGACACCTCGCGGT	
Δ9FAD_HindIII_F	CCCAAGCTTATGGACAAGTTTGCAGAGGAGCAG	construction of expression vector for *S. cerevisiae*
Δ9FAD_AscI_R	TATTGGCGCGCCTCAGACGGACACCTCGCGGT	

### 2.6. Quantitative real-time PCR

Real-time PCR was carried out on CFX96 Touch Real-Time PCR Detection System (Bio-Rad, CA, USA) using SYBR Premix Ex Taq™II (TaKaRa Biotech Co., Dalian, China). The actin gene in *Auxenochlorella pyrenoidosa* was used as an internal standard, amplified with the specific β-Actin_F and β-Actin_R primers. Real-time PCR was conducted following the procedure: 95°C for 30 s before performing 40 cycles of 95°C for 5 s and 60°C for 45 s, and a melting step at 60–95°C. PCR efficiency of each gene was calculated by relative standard curve using sequential dilutions of the cDNA. The 2^−ΔΔCT^ method was applied to calculate the target gene expression.

### 2.7. Functional expression of Δ9FAD in *Escherichia coli* and *Saccharomyces cerevisiae*

The function of Δ9FAD gene of *Auxenochlorella pyrenoidosa* was confirmed through overexpression in both prokaryote (*Escherichia coli*) and eukaryote (*Saccharomyces cerevisiae*). Firstly, the Δ9FAD gene was amplified with the designed primers: Δ9FAD_F and Δ9FAD_R from cDNA of *A. pyrenoidosa* (table 1). Subsequently, the amplified Δ9FAD fragment was subcloned into vector pET-28a with specific primers: Δ9FAD_BamHI_F and Δ9FAD_ HindIII _R (table 1), leading to a plasmid pET28a-Δ9FAD. Constructed pET28a-Δ9FAD and empty vector pET-28a were separately transformed into BL21(DE3), a common expression host in *E. coli*. The single colony was picked up into a tube with 4 ml LB medium, incubated overnight at 220 r.p.m. and 37°C. A millilitre of overnight culture was transferred into a 250 ml Erlenmeyer flask with 100 ml LB medium. After cultivating for about 4 h with the OD_600_ reaching 0.6–0.8, isopropyl-β-D-thiogalactopyranoside (1 mM) was added to induce for 4–6 h. Cells were harvested by centrifugation at 12 000 r.p.m. for 15 min. The fatty acid compositions were analysed as previously described.

Similarly, the amplified Δ9FAD fragment was inserted into a high copy plasmid pRS41H harbouring a quite strong promoter TEF1. Recombinant plasmid was then transformed into *Saccharomyces cerevisiae* BY4741. One colony was picked up in the selected plates with hygromycin (200 mg ml^−1^) and transferred into 15 ml tube containing 4 ml YPD medium, cultivated overnight in 30°C orbital shaker shaking at 200 r.p.m. A millilitre of overnight culture was transferred into a 250 ml Erlenmeyer flask containing 100 ml YPD medium for cultivating for about 72 h. Cells were harvested by centrifugation at 12 000 r.p.m. for 15 min. The fatty acid compositions were analysed as previously described.

## 3. Results and discussion

### 3.1. Dynamic variations of the fatty acid profiles in *Auxenochlorella pyrenoidosa* under different nitrate concentrations

Our previous research suggested C18:1 accumulated in *Auxenochlorella pyrenoidosa* under nitrate absence after cultivation for 14 days, while C18:3 accumulated with nitrate presence [16]. To gain more insights into the fatty acid changes triggered by different nitrate concentrations, dynamic variations of nitrate concentration in the medium, the growth and the fatty acid profiles of *A. pyrenoidosa* under initial nitrate concentrations of 0 g l^−1^ and 1.5 g l^−1^ were investigated.

**Figure 1.**
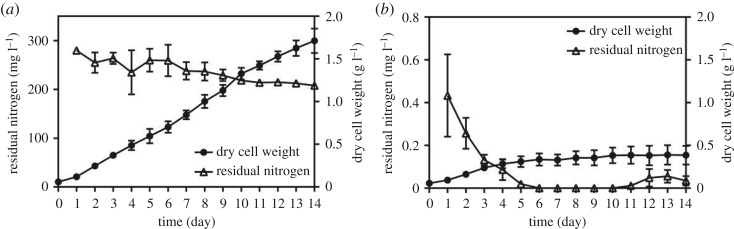
Nitrogen consumption and biomass production of *Auxenochlorella pyrenoidosa* cultivated with initial sodium nitrate concentrations of 1.5 g l^−1^ (*a*) and 0 g l^−1^ (*b*). The residual nitrogen was detected within the whole cultivation by an automatic chemistry analyser. Bars represent means and standard deviation (*n* = 3).

As shown in figure 1*a*, nitrogen concentration was 280 mg l^−1^ in the 1st day in medium containing 1.5 g l^−1^ sodium nitrate initially. Although nitrogen concentration decreased gradually during the cultivation, substantial residual nitrogen was also detected at the end of cultivation. The maximum biomass concentration (1.71 g l^−1^) of *A. pyrenoidosa* under 1.5 g l^−1^ nitrate concentration was obtained after 14 days cultivation. At 0 g l^−1^ initial nitrate concentration, it was remarkable that 0.43 mg l^−1^ nitrogen in the medium was detected until the 5th day (figure 1*b*), owing to trace amounts of nitrate inevitably introduced into the medium during the process of inoculation. As shown in figure 1*b*, the nitrate was completely consumed after 5 days cultivation. However, the biomass concentration continued to increase until the 7th day of cultivation. The biomass concentration of 0.39 g l^−1^ was obtained after 14 days cultivation. It was hypothesized that, after the nitrate in medium was depleted, cells started to use the intracellular nitrogen pool to support the synthesis of cell material for further cell division [34]. On the 12th day, the nitrate concentration slightly increased, this might be due to the nitrogen decomposition of dead algal cells [35].

As can be seen from table 2, the main fatty acid components of *A. pyrenoidosa* were fatty acids with C16 and C18 at 1.5 g l^−1^ nitrate concentration, accounting for over 99.01%. No significant changes were observed in fatty acid profiles during 14 days cultivation. C18:2 and C18:3 made up the majority of C18 fatty acids, accounting for over 20.39% and 23.44%, respectively. The proportion of C18:1 was below 1.12% under 1.5 g l^−1^ nitrate concentration (table 2). At 0 g l^−1^, the fatty acid profiles were similar to that of 1.5 g l^−1^ during the initial 7 days (tables 2 and 3). However, C18:1 significantly accumulated up to 34.40% since the 8th day, whereas C18:3 was not detected (table 3).

**Table 2.** Dynamic variations of the fatty acid profiles in *Auxenochlorella pyrenoidosa* at 1.5 g l^−1^ nitrate concentration. Results are expressed as the mean ± s.d. of three replicates (*n* = 3). n.d., not detected.

fatty acid content (%)												
	C14:0	C16:0	C16:1	C16:2	C16:3	C16:4	C17:0	C18:0	C18:1	C18:2	C18:3	C20–C26
day 1	0.31 ± 0.05	23.85 ± 1.23	0.35 ± 0.61	5.78 ± 0.10	14.88 ± 2.24	0.45 ± 0.78	0.55 ± 0.67	1.71 ± 0.26	0.52 ± 0.90	23.12 ± 2.34	28.43 ± 1.67	n.d.
day 2	0.42 ± 0.16	31.38 ± 5.43	n.d.	2.99 ± 3.85	11.93 ± 3.77	0.44 ± 0.62	0.26 ± 0.11	3.11 ± 1.37	0.88 ± 0.82	22.11 ± 1.54	26.45 ± 3.48	0.10 ± 0.17
day 3	0.31 ± 0.28	27.82 ± 1.13	0.14 ± 0.25	6.75 ± 3.39	14.74 ± 4.44	0.33 ± 0.57	0.13 ± 0.13	2.87 ± 0.31	0.03 ± 0.04	22.23 ± 2.21	24.61 ± 4.13	n.d.
day 4	0.19 ± 0.17	24.01 ± 3.81	0.90 ± 1.55	5.05 ± 1.17	15.78 ± 2.29	0.76 ± 1.32	0.27 ± 1.14	1.70 ± 0.12	n.d.	21.40 ± 0.79	29.88 ± 0.96	n.d.
day 5	0.41 ± 0.12	29.55 ± 6.22	n.d.	3.83 ± 5.10	11.68 ± 3.65	n.d.	0.30 ± 0.03	3.31 ± 1.81	n.d.	27.48 ± 1.19	23.44 ± 2.83	0.05 ± 0.08
day 6	0.34 ± 0.07	27.06 ± 7.00	0.82 ± 1.42	3.75 ± 2.29	13.15 ± 5.01	0.47 ± 0.80	0.36 ± 0.12	2.35 ± 1.44	0.34 ± 0.58	20.92 ± 0.62	30.40 ± 0.97	n.d.
day 7	0.23 ± 0.19	26.82 ± 4.49	0.41 ± 0.70	4.98 ± 1.96	13.73 ± 2.45	n.d.	0.11 ± 0.09	2.42 ± 0.60	0.50 ± 0.86	23.56 ± 1.56	27.23 ± 0.94	n.d.
day 8	0.39 ± 0.07	29.61 ± 4.96	0.28 ± 0.47	3.80 ± 2.37	12.39 ± 5.15	0.10 ± 0.10	0.20 ± 0.22	2.21 ± 1.51	0.58 ± 1.01	20.39 ± 0.75	30.03 ± 0.49	n.d.
day 9	0.39 ± 0.05	32.02 ± 2.04	0.40 ± 0.42	2.12 ± 1.21	9.26 ± 2.99	n.d.	0.36 ± 0.09	3.50 ± 0.99	n.d.	22.11 ± 1.21	29.82 ± 0.93	n.d.
day 10	0.35 ± 0.06	27.18 ± 6.43	n.d.	3.84 ± 2.42	12.98 ± 5.54	n.d.	0.39 ± 0.38	2.60 ± 1.69	0.35 ± 0.61	21.69 ± 0.21	30.60 ± 0.23	n.d.
day 11	0.38 ± 0.04	28.83 ± 4.01	0.02 ± 0.02	1.63 ± 1.88	15.08 ± 9.07	n.d.	0.51 ± 0.32	2.55 ± 1.81	n.d.	22.10 ± 0.97	28.82 ± 3.62	n.d.
day 12	0.38 ± 0.03	28.24 ± 2.46	0.35 ± 0.54	5.62 ± 2.99	14.77 ± 2.90	n.d.	0.33 ± 0.16	2.33 ± 0.25	0.01 ± 0.01	22.67 ± 4.46	25.27 ± 3.54	n.d.
day 13	0.37 ± 0.12	28.89 ± 7.46	0.32 ± 0.55	3.70 ± 4.40	14.90 ± 3.51	n.d.	0.28 ± 0.26	2.04 ± 0.65	1.12 ± 1.10	23.70 ± 3.70	24.61 ± 3.53	n.d.
day 14	0.48 ± 0.08	29.48 ± 4.78	n.d.	2.21 ± 3.82	12.07 ± 2.18	n.d.	0.41 ± 0.15	3.69 ± 2.40	0.48 ± 0.83	25.73 ± 0.60	25.44 ± 1.48	n.d.

**Table 3.** Dynamic variations of the fatty acid profiles in *Auxenochlorella pyrenoidosa* at 0 g l^−1^ nitrate concentration. Results are expressed as the mean ± s.d. of three replicates (*n* = 3). n.d., not detected.

fatty acid Content (%)												
	C14:0	C16:0	C16:1	C16:2	C16:3	C16:4	C17:0	C18:0	C18:1	C18:2	C18:3	C20–C26
day 1	0.32 ± 0.07	23.65 ± 3.07	0.36 ± 0.61	4.93 ± 4.51	15.57 ± 0.85	0.46 ± 0.79	0.18 ± 0.18	1.56 ± 0.45	0.13 ± 0.02	26.69 ± 4.65	26.12 ± 2.00	0.06 ± 0.01
day 2	0.29 ± 0.11	27.92 ± 0.51	0.39 ± 0.66	3.41 ± 2.95	14.42 ± 0.56	0.45 ± 0.78	0.38 ± 0.07	1.85 ± 0.45	0.07 ± 0.10	26.07 ± 7.19	24.75 ± 3.39	n.d.
day 3	0.39 ± 0.12	30.95 ± 7.77	0.47 ± 0.80	4.08 ± 5.02	11.71 ± 3.39	0.51 ± 0.72	0.56 ± 0.40	1.91 ± 0.72	n.d.	24.91 ± 2.28	24.67 ± 3.44	n.d.
day 4	0.38 ± 0.07	28.44 ± 4.32	n.d.	5.96 ± 7.56	10.43 ± 3.79	0.50 ± 0.70	0.61 ± 0.32	3.01 ± 2.34	n.d.	29.82 ± 7.73	21.15 ± 5.63	n.d.
day 5	0.28 ± 0.23	34.98 ± 1.68	n.d.	1.27 ± 1.17	9.43 ± 1.77	n.d.	0.31 ± 0.28	4.31 ± 0.15	0.78 ± 1.09	24.05 ± 6.23	24.87 ± 5.93	n.d.
day 6	0.45 ± 0.04	30.95 ± 1.99	n.d.	2.76 ± 3.79	10.12 ± 3.48	0.21 ± 0.29	0.38 ± 0.34	3.93 ± 2.04	n.d.	25.17 ± 2.73	25.99 ± 4.46	0.12 ± 0.10
day 7	0.46 ± 0.16	35.98 ± 2.89	0.13 ± 0.23	0.96 ± 0.83	8.58 ± 2.65	0.13 ± 0.23	0.46 ± 0.24	2.22 ± 2.29	n.d.	25.76 ± 6.40	25.13 ± 9.51	0.32 ± 0.56
day 8	0.35 ± 0.02	31.37 ± 3.21	0.11 ± 0.19	0.54 ± 0.50	7.05 ± 0.46	1.62 ± 2.55	0.70 ± 0.39	4.88 ± 0.47	34.40 ± 1.95	18.52 ± 1.79	n.d.	0.46 ± 0.37
day 9	0.27 ± 0.12	31.89 ± 2.56	0.23 ± 0.19	0.69 ± 0.60	7.11 ± 0.91	0.23 ± 0.20	0.58 ± 0.26	5.07 ± 0.23	34.73 ± 3.53	18.21 ± 4.75	n.d.	0.95 ± 0.70
day 10	0.32 ± 0.04	33.64 ± 3.94	0.11 ± 0.19	0.83 ± 0.70	6.87 ± 0.40	0.23 ± 0.20	0.71 ± 0.32	4.74 ± 0.34	32.44 ± 2.65	18.70 ± 4.24	n.d.	1.36 ± 1.02
day 11	0.40 ± 0.03	33.63 ± 2.77	0.26 ± 0.23	0.79 ± 0.69	7.22 ± 0.72	0.10 ± 0.17	0.71 ± 0.42	4.76 ± 0.15	28.14 ± 1.87	22.61 ± 1.95	n.d.	1.30 ± 0.77
day 12	0.35 ± 0.12	34.24 ± 1.07	0.22 ± 0.38	0.66 ± 0.60	7.43 ± 1.48	0.07 ± 0.12	0.62 ± 0.40	4.93 ± 0.98	28.30 ± 7.14	22.35 ± 6.20	n.d.	0.79 ± 0.78
day 13	0.27 ± 0.04	34.44 ± 1.08	0.10 ± 0.17	0.80 ± 0.13	5.86 ± 0.44	0.07 ± 0.11	0.44 ± 0.49	5.07 ± 0.40	32.47 ± 2.28	19.51 ± 2.11	n.d.	0.85 ± 1.15
day 14	0.30 ± 0.09	33.87 ± 3.14	0.35 ± 0.00	0.99 ± 0.45	6.22 ± 1.47	0.16 ± 0.21	0.73 ± 0.47	4.67 ± 0.78	30.88 ± 3.42	20.87 ± 3.64	n.d.	0.99 ± 0.90

Based on the results above, it was clear that nitrate had pronounced influence on the composition of C18 fatty acids. The presence of nitrate contributed to the accumulation of C18:3 (figure 1*a* and table 2). However, once there was no residual nitrate, C18:1 accumulated at the expense of C18:3 (figure 1*b* and table 3). These results suggested that nitrate could induce the desaturation of C18 fatty acids.

Similar changes of fatty acids were also observed in many other studies. For example, the proportion of C18:1 in *Chlorococcum oleofaciens* and *Pseudokirchneriella subcapitata* increased accompanied by the decrease of C18:3 under nitrogen-depleted condition after a growth cycle [36]. A gradual decrease in C16:4 and C18:3 as well as an increase in C16:0 and C18:1 were observed in *Chlamydomonas reinhardtii* during 6 days under nitrogen starvation culture [37]. Various microalgae species such as *Pavlova lutheri* [38], *Neochloris oleoabundans* HK-129 [39], *Ankistrodesmus falcatus* and *Chlorella vulgaris* [17] also have the trend of saturation under nitrogen depletion. Compared with the previous research, our results firstly detected the dynamic variations of fatty acids and nitrate concentration during the whole cultivation, and set up the intuitive relationship between the nitrate concentration and fatty acid desaturation. This intuitive relationship could be helpful to regulate the desaturation of fatty acids, which is closely related to the quality of microalgae biodiesel.

### 3.2. Changes of fatty acid composition in *Auxenochlorella pyrenoidosa* upon nitrate replenishment

Interestingly, the change of C18 fatty acid profiles occurred on the 8th day under nitrate absence. To verify if the fatty acid profiles could recover with nitrate addition, the nitrate concentration in the culture was replenished to 1.5 g l^−1^ at this time point.

**Figure 2.**
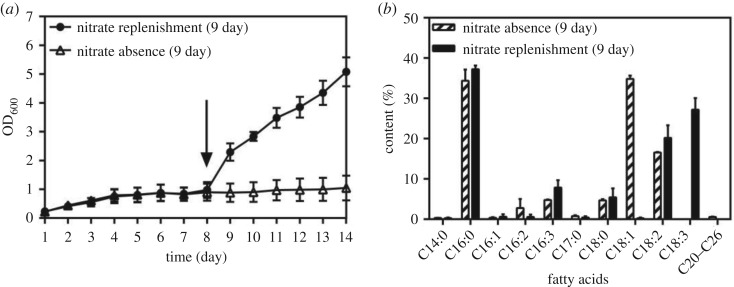
Growth (*a*) and fatty acid composition (*b*) of *Auxenochlorella pyrenoidosa* under nitrate absence and nitrate replenishment cultures. Initially, *A. pyrenoidosa* was cultured at 0 g l^−1^ nitrate concentration. Nitrate replenishment to 1.5 g l^−1^ was performed after 8 days cultivation under nitrate absence. The arrow indicates when nitrate was replenished. Bars represent means and standard deviation (*n* = 3).

The cell growth and fatty acid composition of *A. pyrenoidosa* under nitrate replenishment are described in figure 2. As shown in figure 2*a*, the cells grew slowly under nitrate absence and entered logarithmic phase immediately after the nitrogen was replenished. At the same time, C18 fatty acid composition of *A. pyrenoidosa* changed rapidly within 24 h after replenishment of nitrate (figure 2*b*), while the proportion of other fatty acids remained basically unchanged. C18:3 dramatically accumulated from 0 to 27.20% while C18:1 sharply decreased from 34.79% to 0.22%. After nitrate supplement, the degree of unsaturation of C18 fatty acids increased. These results directly manifested that replenishment of nitrate could stimulate the desaturation of C18 fatty acids in *A. pyrenoidosa*. It was in accordance with the report of *Chlorella* sp. that under 10 days nitrogen starvation, PUFA increased from 26.75 ± 0.45% to 29.99 ± 1.13% within the first day after replenishment of nitrogen [40]. However, this was in contrast to the fatty acid composition changes of *Klebsormidium* sp. LJ1 and *Uronema* sp. that nitrogen starvation induced an increase in PUFA [15], which might be explained as species dependent [16].

C18:1 is favourable for biodiesel quality, since it could balance cold flow properties and oxidation stability [41]. It was worthwhile to mention that *A. pyrenoidosa* had a high percentage of C18:1 (28.14%–34.73%) since the 8th day under nitrate absence (table 3). The biomass of *A. pyrenoidosa* increased rapidly since the 2nd day, and reached a plateau on the 6th day at 0 g l^−1^ nitrate concentration (figure 1*b*). These results suggested the cultivation of 8 days under nitrate absence was a better approach to obtain high quality fatty acid composition without sacrificing production efficiency in *A. pyrenoidosa*.

### 3.3. Expression of fatty acid desaturase genes of *Auxenochlorella pyrenoidosa* under different nitrate concentrations and growth phases

To explain the variations of C18 fatty acid desaturation under different nitrate levels and guide the production of biodiesel, the influence of nitrate on transcriptional levels of relative genes were explored. As mentioned above, the desaturation of C18 fatty acids was catalysed by Δ9, Δ12 and Δ15FADs. Thus real-time PCR was conducted to investigate whether the transcriptional levels of the three FAD genes were correlated with the variations of C18 fatty acid desaturation under different nitrate concentrations.

**Figure 3.**
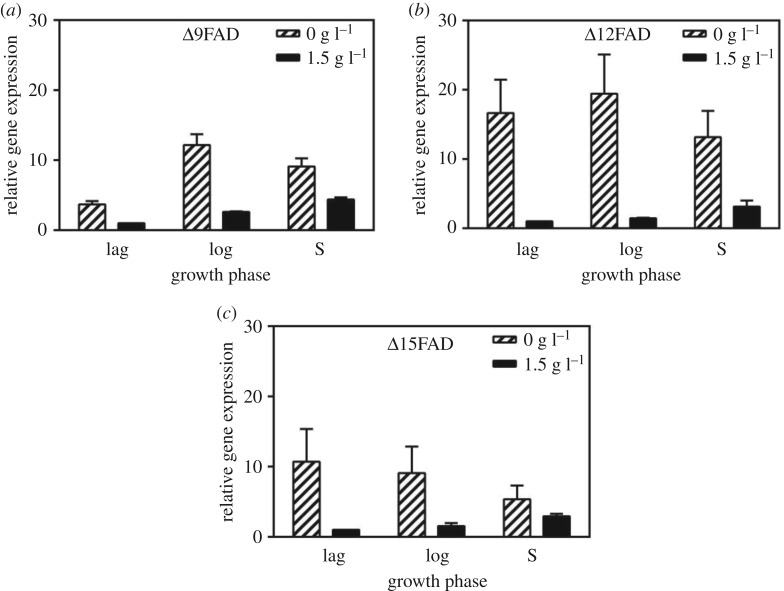
Relative gene expression in response to different nitrate concentrations. The expressions of three genes Δ9FAD (*a*), Δ12FAD (*b*) and Δ15FAD (*c*) in three different growth phases lag (lag), logarithmic (log) and stationary phase (S) were detected by real-time PCR. Algal cells were collected in lag (day 2), logarithmic (day 7) and stationary (day 12) phases under the nitrate concentrations of 0 and 1.5 g l^−1^. Bars represent means and standard deviation (*n* = 3).

The expression levels of the three FADs in lag phase at 1.5 g l^−1^ nitrate concentration were set to 1. As shown in figure 3*a*, the expression of Δ9FAD was significantly upregulated in logarithmic phase at 0 g l^−1^, reaching the highest expression level to about 3.3-fold that in lag phase. The expressions of Δ12FAD and Δ15FAD were increased 16% (figure 3*b*) and decreased 14% (figure 3*c*) in logarithmic phase under 0 g l^−1^, respectively. In stationary phase under nitrate absence, the expression of all the three FADs downregulated 28%, 31% and 38% compared with that in logarithmic phase, respectively. However, the expression levels of the three FADs were all increased slightly from lag to stationary phase at 1.5 g l^−1^. The expression levels of the three FADs were rather lower at 1.5 g l^−1^ than 0 g l^−1^ (figure 3*a–c*).

At 1.5 g l^−1^ nitrate concentration, three FADs upregulated slightly with the gradual consumption of nitrate (figure 1*a*). At 0 g l^−1^, accompanied by the reduction of the nitrate concentration from lag to logarithmic phase (figure 1*b*), the transcriptional levels of Δ9FAD and Δ12FAD were increased and Δ15FAD was slightly decreased. From logarithmic to stationary phase, transcriptional levels of three FADs were decreased with an increase of nitrate concentration (figure 1*b*). The results showed a negative correlation between the transcriptional levels of the three FADs and nitrate concentration.

In logarithmic phase at 0 g l^−1^, the significantly upregulated expression of Δ9FAD was consistent with the accumulation of C18:1 (table 3). The downregulated expression of Δ15FAD coincided with the sharp decrease in C18:3 (table 3). The slightly changed expression of Δ12FAD also led to the stable content of C18:2 (table 3). At 1.5 g l^−1^, the relatively constant low expression levels of the three FADs resulted in the unchanged fatty acid profiles of *A. pyrenoidosa* (table 2).

Indeed, effects of environmental stress on the expressions of fatty acid desaturase genes of microalgae have been widely studied. Low temperature induced the expression of Δ12FAD in *Chlorella vulgaris* [42]. The expressions of Δ4FAD, Δ5FAD and Δ8FAD upregulated in logarithmic growth phase of nitrogen-replete cultures in *Isochrysis* aff. *galbana* [43], thus facilitated the formation of docosapentaenoic acid (DHA). However, to our knowledge, this was the first time that the expressions of three medium chain fatty acid desaturase genes in different nitrate concentrations were reported. The results indicated both the upregulated Δ9FAD expression and the downregulated Δ15FAD expression resulted in the final accumulation of C18:1 and a decrease of C18:3 in logarithmic phase at 0 g l^−1^ nitrate concentration.

### 3.4. Expression of fatty acid desaturase genes of *Auxenochlorella pyrenoidosa* upon nitrate replenishment

To further verify the effect of nitrate concentration on transcriptional levels of the three FADs, the expressions of the three FADs under nitrate replenishment were detected.

**Figure 4.**
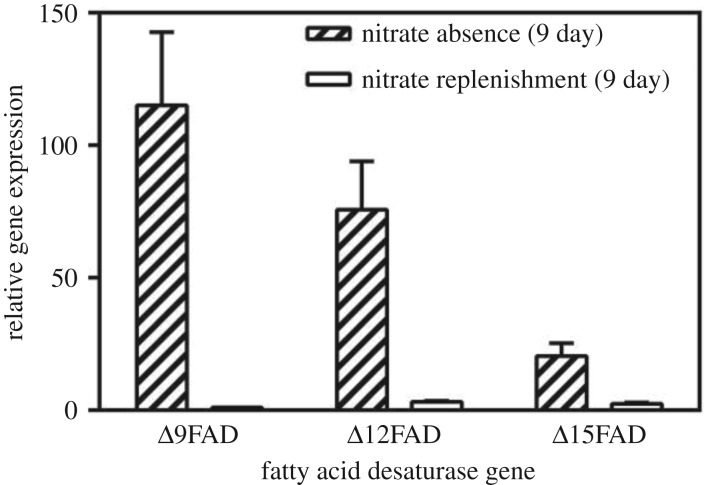
Relative gene expression of Δ9FAD, Δ12FAD and Δ15FAD under nitrate absence and nitrate replenishment. Bars represent means and standard deviation (*n* = 3).

The expression level of Δ9FAD after nitrate replenishment was set to 1. As figure 4 suggests, nitrate replenishment caused a transient reduction in expression levels of all three FADs, which were decreased 99.10%, 95.80% and 88.00%, respectively. This might be owing to the change of carbon flux towards fatty acid synthesis under different nitrate concentrations [44]. Under nitrogen absence, the photosynthetic apparatus of the cells was cannibalized and then redirected to synthesize nitrogen assimilation enzymes, shunting newly fixed carbon toward fatty acid biosynthesis [45,46]. Under nitrate absence, the relative gene expression of Δ9FAD : Δ12FAD : Δ15FAD was 5.6 : 3.7 : 1. It was speculated that Δ9FAD was the key step, high expression level of which led to the accumulation of C18:1 under nitrate absence (figure 4 and table 3). Nitrate replenishment suppressed the expression of three FADs apparently. Of the three FADs, Δ15FAD was the least suppressed one (figure 4). The expression levels of Δ12FAD and Δ15FAD were higher than that of Δ9FAD after nitrate replenishment, therefore increased flux caused the build-up of C18:3.

Our findings suggest the expressions of three FADs in *A. pyrenoidosa* were regulated differentially in response to nitrate stress. Δ15FAD was crucial to C18:3 after nitrate resupply. Δ9FAD was more sensitive to nitrate absence and played a pivotal role in the fatty acid desaturation metabolic pathway under nitrate absence. Besides nitrogen stress, Δ9FAD also had strong responses to other environmental factors. For example, temperature shift [47], salt stress [48] and low light intensities [26] also stimulated the expression of Δ9FAD.

The present study built a relationship among nitrate concentration, expression level of Δ9FAD and fatty acid desaturation in *A. pyrenoidosa*. At 1.5 g l^−1^ nitrate concentration, C18:2 and C18:3 made up the majority in C18 fatty acids (table 2). The degree of fatty acid desaturation in *A. pyrenoidosa* decreased sharply under nitrate absence (table 3). The transcriptional level of Δ9FAD had significantly negative correlation with nitrate. At 1.5 g l^−1^ nitrate concentration, the expression of Δ9FAD upregulated slightly with the gradual consumption of nitrate (figures 1*a* and 3*a*). At 0 g l^−1^, accompanied by the reduction of the nitrate concentration from lag to logarithmic phase (figure 1*b*), the transcriptional level of Δ9FAD was increased significantly, which was consistent with the accumulation of C18:1 (table 3). This correlation could be illustrated more clearly by nitrate replenishment (figures 2*b* and 4). As shown in figure 4, nitrate replenishment caused a sharp downregulation of Δ9FAD to 1% of the nitrate absence level followed by a sharp reduction of C18:1 from 34.79% to 0.22% (figure 2*b*).

However, how the nitrate concentration affected the expression of Δ9FAD was little known. Some pivotal mechanism such as transcription factor and signal transduction pathway remained to be revealed. The incoming technologies such as transcriptome and proteomics analysis provided a useful way to solve this problem. The omics technologies show that nitrogen starvation induced a global stress response in microalgae. Metabolism including nitrogen, amino acids, proteins and carbohydrates, photosynthesis and chlorophyll biosynthesis would be affected [49,50]. In *Chlamydomonas*, the transcriptome research found that nitrogen starvation induced the accumulation of TAG through affecting three genes encoding acyltransferases. Meanwhile, a candidate of transcription factor named nitrogen response regulator (*NRR1*) was also identified, which was closely related to acyltransferases [51]. In *Chlorella vulgaris*, except for the transcription factor, a signal transduction regulator ctrl1 was also identified and proved to be associated with the lipid accumulation [52]. We expect to use the omics technology to elucidate the mechanism of how nitrogen starvation affecting the gene expression in the future.

### 3.5. Overexpression of Δ9FAD resulted in an increase in oleic acid content

**Figure 5.**
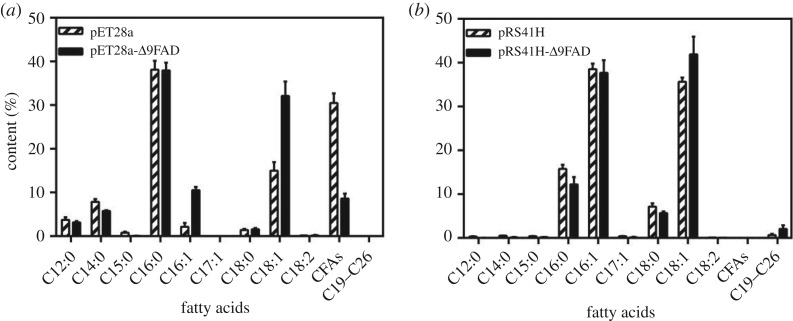
Fatty acid profiles analysed by gas chromatography (GC). *Escherichia coli* cells (*a*) and *Saccharomyces cerevisiae* cells (*b*) with Δ9FAD overexpressed. CFAs: cyclopropane fatty acids, including C17 and C19 cyclopropane fatty acids. Bars represent means and standard deviation (*n* = 3).

In order to further verify the function of Δ9FAD, it was overexpressed in *Escherichia coli* BL21 (DE3) and *Saccharomyces cerevisiae* BY4741. As can be seen from figure 5*a*, after being transformed with pET28a-Δ9FAD, the content of C16:1 and C18:1 in *E. coli* BL21 increased from 2.15% and 14.99% to 10.53% and 32.01%, respectively. It was demonstrated that a functional enzyme encoded by Δ9FAD gene recognized two substrates, C16:0 and C18:0. The content of cyclopropane fatty acids in wild-type strain was very high [53], and the decrease in cyclopropane fatty acids might be due to the redirection of carbon flux caused by overexpression of gene [54]. The results in BL21 had confirmed Δ9FAD's ability of desaturating C18:0, indicating the induced expression of Δ9FAD was not toxic to *E. coli*. Thus a constitutive expression manner was tried in eukaryotic *S. cerevisiae*. Similarly, in *S. cerevisiae* BY4741 transformed with pRS41H-Δ9FAD, the contents of C18:1 increased from 35.65% to 41.88% (figure 5*b*). The relatively slight increase of C18:1 in *S. cerevisiae* transformants could be explained by the high content of C18:1 in original strain, which caused the lower conversion efficiency of Δ9FAD in *S. cerevisiae* compared with that in *E. coli* (figure 5*a*,*b*). It was also possible that the constitutive expression was relatively low. An inductive promoter would be considered for use in the future. Results in both strains showed the enhanced desaturase conversion efficiency, as the content of MUFA increased more or less. Our study further testified the crucial function of Δ9FAD in the C18 fatty acid desaturation metabolic pathways.

The genetic engineering of key enzymes in fatty acid biosynthesis pathways has been used to improve the quantity and quality of oil [55,56]. The content of eicosapentaenoic acid (EPA) content in *Phaeodactylum tricornutum* was enhanced by overexpression of endogenous delta-6 FAD gene with fcpA promoter [57]. The function of Δ5FAD and Δ12FAD in *Nannochloropsis oceanica* CCMP1779 was determined, overexpression of which increased the content of EPA [58]. The overexpression of the Δ12FAD driven by a stress-inducible promoter LDSP in *N. oceanica* caused an increase of C18:2 [59]. Although gene overexpression in some microalgae species have been achieved such as *P. tricornutum* [60,61], *Thalassiosira pseudonana* [62], *Dunaliella* [63] and *Chlamydomonas reinhardtii* [64], the genetic manipulation of *A. pyrenoidosa* for recombinant protein production has not been well established. Taken together, endogenous Δ9FAD gene could be a good candidate for use in future trials aiming to accumulate C18:1 in *A. pyrenoidosa* without accepting a productivity decline.

## 4. Conclusion

The present study established the correlation among nitrate concentration, expression level of Δ9FAD and fatty acid desaturation in *A. pyrenoidosa*. The accumulation of C18:1 under nitrate absence was due to the upregulated expression of Δ9FAD. The transcriptional level of Δ9FAD had negative correlation with nitrate. The results showed Δ9FAD played a crucial role in C18 fatty acid desaturation. Overexpression of Δ9FAD in *E. coli* BL21 and *S. cerevisiae* BY4741 caused an increase in C18:1 of 113.54% and 17.47%, respectively. The establishment of this correlation helps to further understand the mechanism of the fatty acid desaturation metabolism. What's more, the integration of nitrogen absence and genetic engineering could not only help drive forward changing the fatty acid profiles but also make *A. pyrenoidosa* as the microalgae synthetic biology chassis to produce preferred fatty acids for the improvement of biodiesel quality and industrialization of high-valued chemicals.

## Supplementary Material

Alignment of the deduced amino acid sequences

## Supplementary Material

Original data of nitrogen consumption and biomass production of Auxenochlorella pyrenoidosa (figure 1)

## Supplementary Material

Original data of Growth (a) and fatty acid composition (b) of Auxenochlorella pyrenoidosa (figure 2)

## Supplementary Material

Original data of relative gene expression of Δ9FAD, Δ12FAD and Δ15FAD (figure 3 and figure 4)

## Supplementary Material

Original data of Fatty acid profiles analyzed by gas chromatography (GC) (figure 5)

## Data accessibility

Additional supporting information may be found in the electronic supplementary material. Electronic supplementary material, figure S1: Alignment of public database and the deduced amino acid sequences of Δ9, Δ12 and Δ15 fatty acid desaturase enzyme in *A. pyrenoidosa.* The datasets supporting this article have been uploaded as part of the electronic supplementary material.
