# Supplementary material for: Variation of fatty acid desaturation in response to different nitrate levels in Auxenochlorella pyrenoidosa
Source: R Soc Open Sci. 2018 Nov 28;5(11):181236. doi: 10.1098/rsos.181236 (PMC6281909; doi:10.1098/rsos.181236)
Supplement: Alignment of the deduced amino acid sequences [file rsos181236supp1.pdf]

<Supporting Information>

Variation of fatty acid desaturation in response to different nitrate levels in *Auxenochlorella pyrenoidosa*

Qi Zhang,[ab] Zaizhi You [ab] and Xiaoling Miao*[ab]

[a] State Key Laboratory of Microbial Metabolism, School of Life Sciences & Biotechnology, Shanghai Jiao Tong University, 800 Dongchuan Road, Shanghai 200240, China

[b] Biomass Energy Research Center, Shanghai Jiao Tong University, Shanghai 200240, China

*Corresponding author: Xiaoling Miao

Tel: +86-21-34207028; Fax: +86-21-34207028

E-mail: miaoxiaoling@sjtu.edu.cn

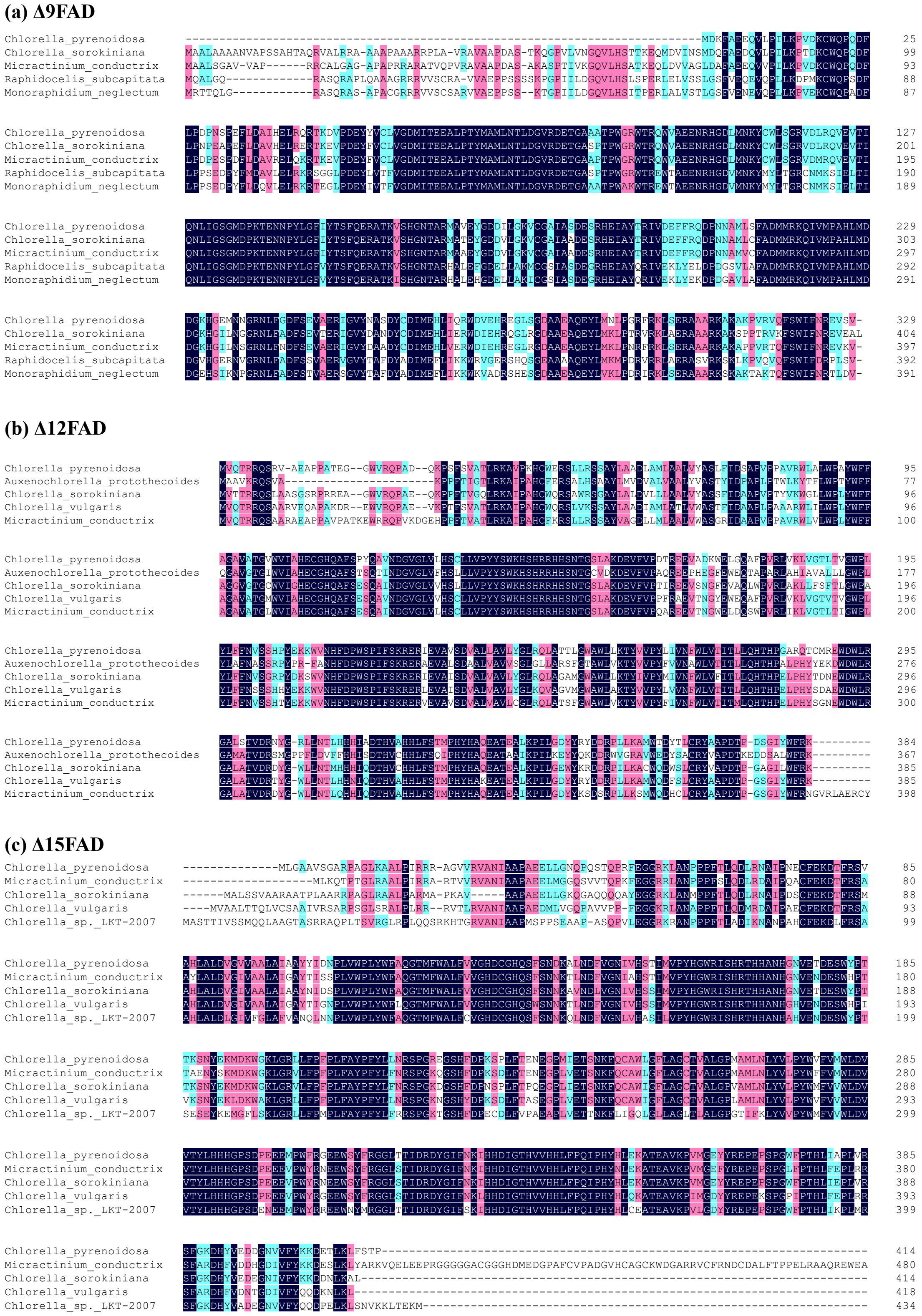

Figure S1. Alignment of the deduced amino acid sequences of the Δ9, Δ12 and Δ15 fatty acid desaturase enzyme from *Auxenochlorella pyrenoidosa* (KQ481983.1), *Chlorella vulgaris* (ACF98528.1, BAB78717.1), *Micractinium conductrix* (PSC67766.1, PSC74767.1, PSC71199.1), *Chlorella sorokiniana* (PRW44938.1, PRW57598.1, PRW60085.1), *Monoraphidium neglectum* (XP_013899493.1), *Raphidocelis subcapitata* (GBF93822.1), *Auxenochlorella protothecoides* (XP_011396739.1) and *Chlorella* sp. LKT-2007 (ABU54076.2).
