# Supplementary material for: Variation of fatty acid desaturation in response to different nitrate levels in Auxenochlorella pyrenoidosa
Source: R Soc Open Sci. 2018 Nov 28;5(11):181236. doi: 10.1098/rsos.181236 (PMC6281909; doi:10.1098/rsos.181236)
Supplement: Original data of nitrogen consumption and biomass production of Auxenochlorella pyrenoidosa (figure 1) [file rsos181236supp2.pdf]

Nitrogen consumption and biomass production of *Auxenochlorella pyrenoidosa* cultivated with different sodium nitrate concentrations of 1.5 g $L^{-1}$ (a) and 0 g $L^{-1}$ (b).

Residual nitrogen in the medium of *Auxenochlorella pyrenoidosa*.

| | 0 g $L^{-1}$ | | | 1.5 g $L^{-1}$ | | |
|---|---|---|---|---|---|---|
| 1 | 0.350 | 0.150 | 0.800 | 289.5049 | 271.0400 | 278.8000 |
| 2 | 0.240 | 0.140 | 0.390 | 284.8275 | 265.2000 | 214.6300 |
| 3 | 0.115 | 0.180 | 0.100 | 283.7324 | 264.3000 | 243.4300 |
| 4 | 0.090 | 0.170 | 0.000 | 282.9538 | 277.4000 | 144.3800 |
| 5 | 0.030 | 0.030 | 0.000 | 280.4270 | 286.0000 | 212.9200 |
| 6 | 0.000 | 0.000 | 0.000 | 277.3757 | 303.8000 | 196.5800 |
| 7 | 0.000 | 0.000 | 0.000 | 232.8758 | 271.4000 | 209.6400 |
| 8 | 0.000 | 0.000 | 0.000 | 227.6463 | 272.4000 | 210.3100 |
| 9 | 0.000 | 0.000 | 0.000 | 226.2052 | 250.4300 | 210.0100 |
| 10 | 0.000 | 0.000 | 0.000 | 224.1224 | 220.4500 | 210.6000 |
| 11 | 0.000 | 0.036 | 0.000 | 219.8219 | 210.0000 | 211.6800 |
| 12 | 0.130 | 0.016 | 0.000 | 219.0323 | 215.6700 | 210.0000 |
| 13 | 0.100 | 0.066 | 0.000 | 217.9032 | 218.2300 | 201.0000 |
| 14 | 0.030 | 0.074 | 0.000 | 203.2258 | 220.2100 | 200.0000 |

Biomass production of *Auxenochlorella pyrenoidosa*.

| | 0 g $L^{-1}$ | | | 1.5 g $L^{-1}$ | | |
|---|---|---|---|---|---|---|
| | 0.057000 | 0.0574494 | 0.056500 | 0.0545514 | 0.056580 | 0.065000 |
| 1 | 0.076000 | 0.0872988 | 0.117438 | 0.0815028 | 0.149316 | 0.128450 |
| 2 | 0.154532 | 0.1745286 | 0.160618 | 0.229011 | 0.2632074 | 0.243211 |
| 3 | 0.267554 | 0.2646564 | 0.179745 | 0.408687 | 0.3278328 | 0.377099 |
| 4 | 0.263207 | 0.3805764 | 0.210754 | 0.5819874 | 0.3924582 | 0.487802 |
| 5 | 0.333629 | 0.405789 | 0.192786 | 0.7497816 | 0.4570836 | 0.582857 |
| 6 | 0.356523 | 0.4460712 | 0.204088 | 0.8089008 | 0.579669 | 0.718483 |
| 7 | 0.392748 | 0.397095 | 0.199451 | 0.9132288 | 0.743406 | 0.875265 |
| 8 | 0.401442 | 0.4588224 | 0.204378 | 1.090007 | 0.8483136 | 1.069431 |
| 9 | 0.419410 | 0.4657776 | 0.178586 | 1.143330 | 1.010022 | 1.237515 |
| 10 | 0.457953 | 0.488382 | 0.201770 | 1.247078 | 1.267364 | 1.465588 |
| 11 | 0.460561 | 0.5118558 | 0.186121 | 1.358651 | 1.389660 | 1.519490 |
| 12 | 0.448390 | 0.5379378 | 0.158879 | 1.460081 | 1.476600 | 1.650000 |
| 13 | 0.450708 | 0.5472114 | 0.170000 | 1.500364 | 1.575132 | 1.801176 |
| 14 | 0.427524 | 0.550000 | 0.180000 | 1.560000 | 1.580000 | 1.996791 |
