# Supplementary material for: Variation of fatty acid desaturation in response to different nitrate levels in Auxenochlorella pyrenoidosa
Source: R Soc Open Sci. 2018 Nov 28;5(11):181236. doi: 10.1098/rsos.181236 (PMC6281909; doi:10.1098/rsos.181236)
Supplement: Original data of Growth (a) and fatty acid composition (b) of Auxenochlorella pyrenoidosa (figure 2) [file rsos181236supp3.pdf]

Growth (a) and fatty acid composition (b) of *Auxenochlorella pyrenoidosa* under nitrate absence and nitrate replenishment cultures.

Growth

	Nitrate absence			Nitrate replenishment		
1.	0.206	0.273	0.200	0.220	0.260	0.206
2.	0.507	0.360	0.459	0.322	0.403	0.507
3.	0.818	0.481	0.525	0.314	0.517	0.818
4.	1.218	0.520	0.632	0.480	0.5584	1.218
5.	1.305	0.560	0.570	0.497	0.598	1.305
6.	1.444	0.590	0.607	0.520	0.653	1.444
7.	1.275	0.607	0.593	0.530	0.719	1.275
8.	1.488	0.593	0.610	0.550	0.885	1.488
9.	1.512	0.610	0.521	1.954	2.028	2.893
10.	1.590	0.521	0.601	2.860	2.552	3.093
11.	1.671	0.601	0.635	4.140	2.980	3.325
12.	1.761	0.583	0.598	4.560	3.390	3.603
13.	1.793	0.590	0.603	5.136	4.200	3.707
14.	1.903	0.648	0.584	5.964	5.040	4.224

fatty acid composition

	Nitrate absence			Nitrate replenishment		
C14:0	0.27	0.28	0.369061	0.00	0.29	0.536344
C16:0	37.69	36.58	28.75454	37.93	35.40	38.36301
C16:1	0.38	0.64	0.00	1.82	0.00	0.00
C16:2	0.54	0.53	7.24042	0.00	1.72	0.00
C16:3	4.69	5.03	4.564922	4.31	8.90	10.36323
C17:0	0.62	0.58	1.148675	0.00	0.22	1.039892
C18:0	4.48	5.28	4.353405	9.47	4.97	1.816237
C18:1	34.25	33.67	36.4594	0.00	0.68	0.00
C18:2	16.33	16.61	16.7614	17.21	16.94	26.44118
C18:3	0.00	0.00	0.00	29.23	30.85	21.5314
C20-C26	0.52	0.76	0.354171	0.00	0.00	0.00
