# Supplementary material for: Variation of fatty acid desaturation in response to different nitrate levels in Auxenochlorella pyrenoidosa
Source: R Soc Open Sci. 2018 Nov 28;5(11):181236. doi: 10.1098/rsos.181236 (PMC6281909; doi:10.1098/rsos.181236)
Supplement: Original data of relative gene expression of Δ9FAD, Δ12FAD and Δ15FAD (figure 3 and figure 4) [file rsos181236supp4.pdf]

1. Relative gene expression of Δ9FAD, Δ12FAD and Δ15FAD under nitrate absence and nitrate replenishment

	Nitrate absence			Nitrate replenishment		
delta9	147	99	99	1	1	1
delta12	96.7	65.1	65.1	3.2	2.7	3.6
delta15	26.1	17.6	17.6	2.9	1.95	2.5

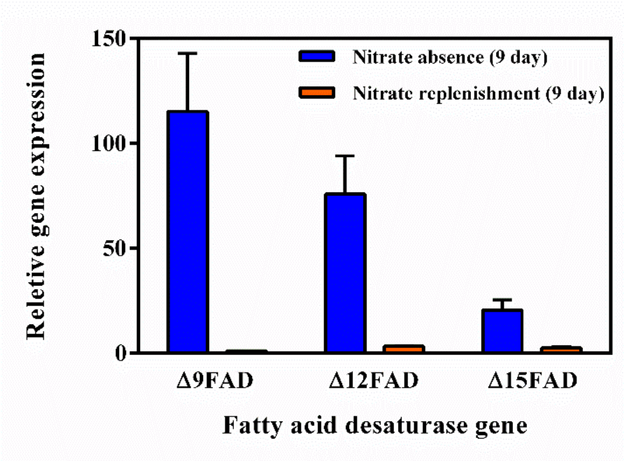

2. Relative gene expression in response to different nitrate concentrations. The expressions of three genes Δ9FAD (a), Δ12FAD (b) and Δ15FAD (c) in three different growth phases lag (Lag), logarithmic (Log) and stationary phase (S) were detected by real time PCR.

Delta 9 FAD

	$0\ g\ L^{-1}$			$1.5\ g\ L^{-1}$		
lag	1	1	1	0.28	0.22	0.34
logarithmic	3.07	3.39	3.44	0.73	0.57	0.886
stationary	3.458	2.034	1.85	1.22	0.96	1.49

Delta 12 FAD

	$0\ g\ L^{-1}$			$1.5\ g\ L^{-1}$		
lag	1	1	1	0.1207	0.04	0.06
logarithmic	1.301	1.11	1.09	0.178	0.059	0.08805
stationary	0.666	0.965	0.75	0.37	0.125	0.188

Delta 15FAD

	$0\ g\ L^{-1}$			$1.5\ g\ L^{-1}$		
lag	1	1	1	0.148	0.186	0.05
logarithmic	0.927	0.83	0.841	0.2294	0.2883	0.0775
stationary	0.66	0.48	0.463	0.43	0.54	0.14

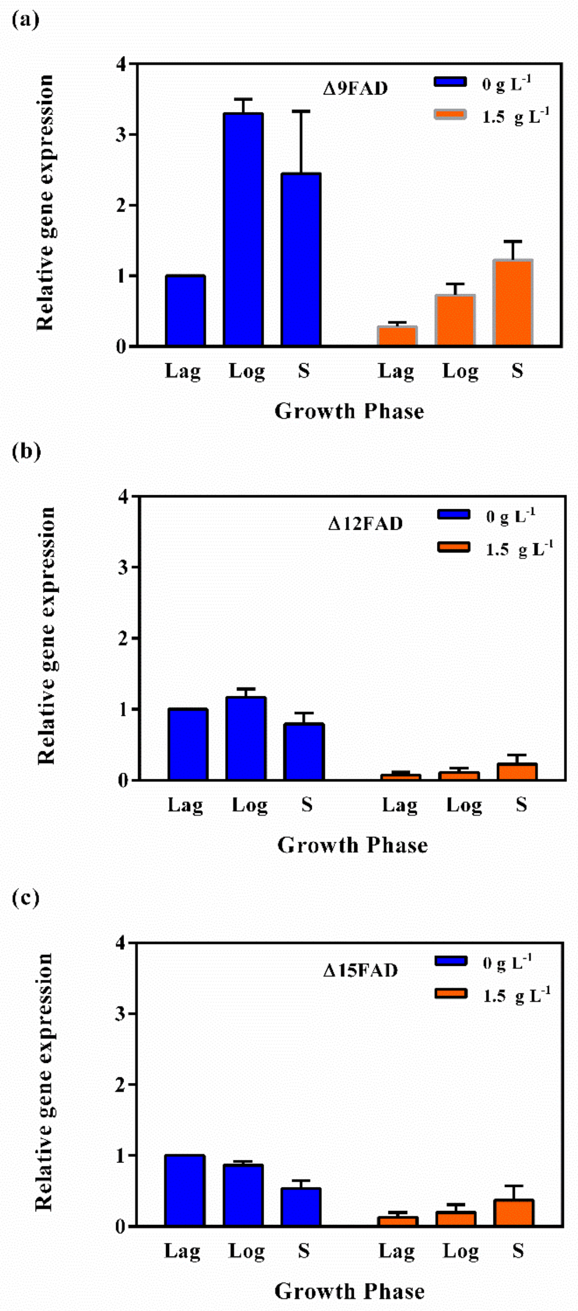
(a)
Δ9FAD
0 g L-1
1.5 g L-1
Relative gene expression
0
1
2
3
4
Lag
Log
S
Lag
Log
S
Growth Phase
(b)
Δ12FAD
0 g L-1
1.5 g L-1
Relative gene expression
0
1
2
3
4
Lag
Log
S
Lag
Log
S
Growth Phase
(c)
Δ15FAD
0 g L-1
1.5 g L-1
Relative gene expression
0
1
2
3
4
Lag
Log
S
Lag
Log
S
Growth Phase
