# Supplementary material for: Variation of fatty acid desaturation in response to different nitrate levels in Auxenochlorella pyrenoidosa
Source: R Soc Open Sci. 2018 Nov 28;5(11):181236. doi: 10.1098/rsos.181236 (PMC6281909; doi:10.1098/rsos.181236)
Supplement: Original data of Fatty acid profiles analyzed by gas chromatography (GC) (figure 5) [file rsos181236supp5.pdf]

Fatty acid profiles analyzed by gas chromatography (GC). *Escherichia coli* cells (a) and *Saccharomyces cerevisiae* cells (b) with Δ9FAD overexpressed.

Escherichia coli

	pET-28a			pET28a-Δ9FAD		
C12:0	4.410000	3.228237	3.490821	2.832607	3.197542	3.422020
C14:0	8.590000	7.376029	7.535871	5.624025	5.939918	5.703921
C15:0	0.460000	0.826011	0.973984	0.061955	0.102412	0.070891
C16:0	35.730000	39.458210	39.107560	39.629420	38.127110	36.117300
C16:1	3.140000	1.705691	1.616060	10.597290	11.220070	9.769495
C17:0	0.480000	0.000000	0.000000	0.129647	0.118116	0.075321
C17:0 cyclo	19.270000	19.284460	19.574390	8.236806	6.661584	6.651639
C18:0	1.610000	1.432595	1.084018	1.850505	1.595699	1.243465
C18:1	17.160000	14.353310	13.459510	29.157990	31.451750	35.688300
C18:2	0.150000	0.121131	0.113864	0.231069	0.209035	0.000000
C19 cyclo	8.960000	12.219200	13.047640	1.651216	1.377219	1.260634
C12:0	4.410000	3.228237	3.490821	2.832607	3.197542	3.422020
C14:0	8.590000	7.376029	7.535871	5.624025	5.939918	5.703921
C15:0	0.460000	0.826011	0.973984	0.061955	0.102412	0.070891
C16:0	35.730000	39.458210	39.107560	39.629420	38.127110	36.117300

Saccharomyces cerevisiae

	pRS41H			pRS41H-Δ9FAD		
C12:0	0.44	0.194721	0.292694	0.10	0.00	0.00
C14:0	0.57	0.530074	0.461556	0.09	0.086751	0.282762
C15:0	0.34	0.40026	0.439041	0.11	0.113603	0.304513
C16:0	14.99	14.80961	17.55038	12.41	9.231476	14.99728
C16:1	36.62	41.00	38.00	38.46	32.40	42.22
C17:1	0.43	0.367806	0.405268	0.12	0.00	0.358891
C18:0	8.59	6.436608	6.473038	5.02	5.710096	6.329527
C18:1	37.53	34.62787	34.7968	41.35	49.18449	35.10
C18:2	0.08	0.108178	0.123832	0.03	0.00	0.097879
CFAs	0.00	0.00	0.00	0.00	0.00	0.00
C19-C26	0.18	0.670705	1.137003	2.17	3.455284	0.554649
